# Evaluation of Behavior of Castable versus Machined Solid Abutments for Morse Tapper Implant Connection: A Clinical Retrospective Study

**DOI:** 10.3390/medicina59071250

**Published:** 2023-07-05

**Authors:** Sergio Alexandre Gehrke, Antonio Scarano, Guillermo Castro Cortellari, Gustavo Vicentis Oliveira Fernandes, Sidney Eiji Watinaga, Marco Aurélio Bianchini

**Affiliations:** 1 Department of Research, Bioface/PgO/UCAM, Calle Cuareim 1483, Montevideo 11100, Uruguay; ascarano@unich.it (A.S.); guile237@gmail.com (G.C.C.); 2 Instituto de Bioingenieria, Universidad Miguel Hernández, Avda. Ferrocarril s/n, 03202 Elche, Spain; 3 Department of Biotechnology, Universidad Católica de Murcia (UCAM), 30107 Murcia, Spain; 4 Department of Materials Engineering, Pontificia Universidade Católica do Rio Grande do Sul, Porto Alegre 90619-900, Brazil; 5 Department of Innovative Technologies in Medicine & Dentistry, University of Chieti-Pescara, 66100 Chieti, Italy; 6 Periodontics and Oral Medicine Department, University of Michigan School of Dentistry, Ann Arbor, MI 48104, USA; 7 Department of Implantology, Paulista University (UNIP), São Paulo 01311-000, Brazil; sidneywatinaga@hotmail.com; 8 Post-Graduate Program in Implant Dentistry (PPGO), Federal University of Santa Catarina (UFSC), Florianópolis 88040-900, Brazil; bian07@yahoo.com.br

**Keywords:** dental implants, morse taper connection, castable UCLA abutment, machined solid abutments, marginal bone loss

## Abstract

*Objective*: The primary objective of the present retrospective clinical study was to evaluate and compare the clinical performance presented by castable abutments developed for the MT system versus intermediate machined abutments, specifically regarding prosthetic or implant fractures/loss; the secondary objective was to verify the looseness of the abutments and the behavior of the peri-implant soft tissues. *Methods*: This clinical retrospective study was conducted on patients rehabilitated between 2019 and 2020. Inclusion criteria were patients in good general health, with an implants-supporting single crown; with solid machined abutments (control group) or castable UCLA abutments; with a connection portion (base) machined in cobalt-chrome (test group) over Morse taper DuoCone implants in the posterior mandible area; and at least two years in function. Clinical assessment was carried out by the same professional, considering the following parameters: (A) prosthetic: (i) loosening of the fixation screw, (ii) fracture of the screw and (iii) the number of times the patient had some type of complication after the installation of the prostheses were evaluated; (B) biological: (i) without keratinized mucosa (KM), (ii) 1 mm or less, (iii) between 1 and 2 mm and (iv) greater than 2 mm of KM width; and the presence or absence of mucositis. Furthermore, radiographic evaluation was performed in order to assess the marginal bone loss. These evaluations permitted to compare the groups analyzed and patients enrolled. Data were statistically analyzed, with the level of significance set at α = 0.05. *Results*: 79 patients with 120 MT implants were evaluated (80 castable UCLA abutments and 40 machined solid abutments). The follow-up was from 2 to 4 years. There was a 100% implant survival rate. Therefore, the control group showed two fractured abutments (5%) and no abutment loosening (95% for prosthetic survival rate), whereas the test group showed no abutment fracture but nine loosening screws (11.3%) (100% for prosthetic survival rate). Keratinized mucosa was considered thin or absent in 19 implants in the control group (47.5%) and 42 in the test group (52.5%). Mucositis was found in 11 implants in the control group (27.5%) and 27 in the test group (33.8%). A positive correlation was observed between the width of keratinized mucosa and mucositis (r = 0.521, *p* = 0.002). The mean marginal bone loss was 2.3 mm, ranging from 1.1 to 5.8 mm. No correlation was observed when considering marginal bone loss versus the three parameters (implant diameter, implant length and time of the prosthesis in function). *Conclusions*: The results suggest that UCLA-type abutments are a viable option for rehabilitating implants with Morse taper connections, suggesting lower fracture risk. Further research is necessary to confirm these findings and thoroughly evaluate the clinical performance and long-term outcomes.

## 1. Introduction

The predictability of treatment with osseointegrated dental implants has generated a considerable amount of research and investment to obtain predictable long-term rehabilitation, considering the increase in patients’ longevity [1,2]. Several modifications in the implants’ micro- and macro-geometry, as well as in surgical placement techniques, have been proposed in recent decades. This fact pursues to benefit and/or maintain the health of peri-implant tissues [3,4,5]. Additionally, intermediate abutments have undergone several modifications in their designs and surface characteristics to maintain the health of these tissues in the long term [6,7].

The morphology of the implant/abutment (IA) connection and their micro- and macro-design can directly influence the behavior of peri-implant tissues [8,9]. On the other hand, the tension dissipation generated during the loading of the IA set can lead to several undesired consequences, depending on the type of connection. These consequences may include screw loosening and fracture or damage to the implant itself, as well as to other structures in the system [9,10]. Additionally, possible biological complications may occur, such as mucositis, peri-implantitis, marginal bone loss and implant loss [9,11].

Machined intermediate abutments are pieces that serve as a base for the seating of prosthetic crowns on implants. The use of this type of abutment is highly recommended for mechanical and biological reasons, as its degree of adaptation (precision) and biocompatibility (material used for its manufacture) are fundamental for the longevity of rehabilitation treatments on implants [12]. In this sense, several in-vitro and in-vivo studies have been published showing the benefits regarding the behavior of this type of abutment [10,12,13]. However, other manufacturing methods are used, such as milling (CAD/CAM system) and casting, both presenting negative differences (degree of precision and biocompatibility) concerning machined abutments [13].

The universal castable long abutment (UCLA) abutments, developed in 1988 by Lewis et al. [14], elaborate on calcined intermediate abutments. These UCLA abutments were proposed as an alternative to the existing prefabricated intermediate abutments, developed for prostheses on single or multiple implants, for clinical situations with low interocclusal height, decreased mesiodistal distance and malpositioned implants. Currently, the industry has introduced castable abutments with a machined base in chromium/cobalt to improve the precision of the interface between the IA sets.

Morse taper (MT) connection implants, due to their biomechanical characteristics and advantages over hexagonal implants, have been frequently used for all types of rehabilitation (single and multiple, anterior and posterior) [10,15,16]. MT connection fundamentally depends on the fit’s accuracy between the implant’s internal and abutment cone. However, with the increased clinical use of this type of AI sets (MT connection), problems such as those with other implant connections began with MT implants, such as implants placed in areas with reduced inter-occlusal space. For this reason, castable abutments also had to be developed for the MT system. However, only a few behavioral clinical studies exist in the literature on this type of UCLA abutment for the MT connection.

Thus, the primary objective of the present retrospective clinical study was to evaluate and compare the clinical performance presented by castable abutments developed for the MT system versus intermediate machined abutments, specifically regarding prosthetic or implant fractures/loss. The secondary objective was to verify the looseness of the abutments and the behavior of the peri-implant soft tissues. The null hypothesis was that castable abutments might have a similar clinical performance to machined abutments.

## 2. Materials and Methods

This clinical retrospective study was conducted on patients rehabilitated between 2019 and 2021 at CEPID (Center of Research in Dental Implants) of the Health Sciences Center of the UFSC (Universidade Federal de Santa Catarina, Florianopolis, Brazil). The study was approved by the Ethics Committee on Human Research of the UFSC (number 3,490,963—Florianopolis, Brazil). After receiving explanations about each step of the procedures, patients who agreed to participate in the study signed the Free and Informed Consent Term following the Declaration of Helsinki (1975, updated 2013).

### 2.1. Eligibility Criteria

Inclusion criteria were patients in good general health, with an implants-supporting single crown; with solid machined Ideale abutments (control group) or castable UCLA abutments; with a connection portion (base) machined in cobalt-chrome (test group) over Morse taper DuoCone implants in the posterior mandible area; and at least two years in function. All implants included were installed in healed alveolar bone areas, that is, no implants installed in post-extraction sockets were included in the present study. All implants and abutments evaluated belonged to Implacil De Bortoli (São Paulo, Brazil). Solid Morse taper abutments are considered the gold standard for this type of connection [17], justifying their selection as a control group in the present study. All implants included did not receive immediate loading, and were rehabilitated after 60 days post-installation. Furthermore, only implants placed in the posterior area of the mandible (premolars and molars) were enrolled. This excluded patients who did not present data in clinical records or those with local or systemic diseases that compromised clinical analysis.

**Figure 1:**
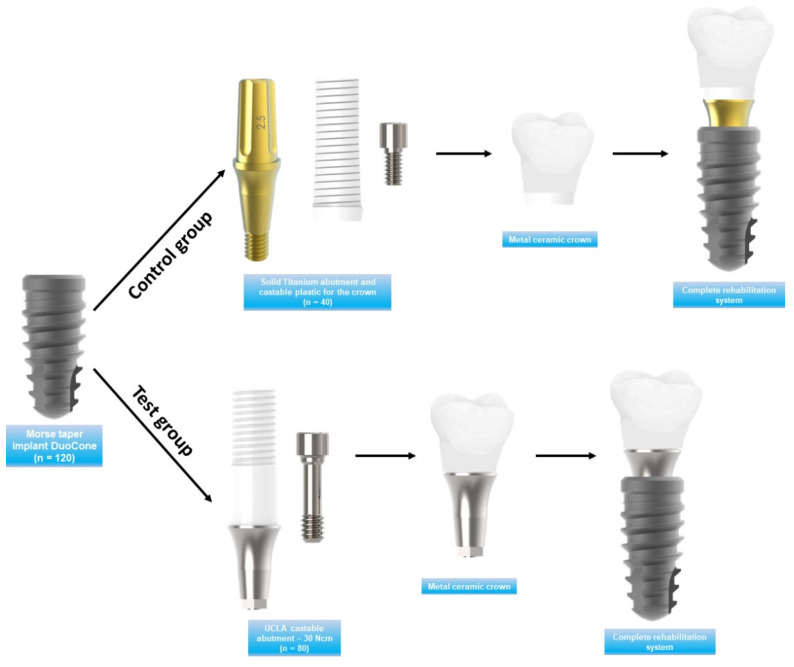
Control and test groups with representative images of the materials used.

After evaluating the inclusion and exclusion criteria, the patients were called for clinical evaluations considering radiographic examination. Figure 1 shows a representative image of the implant and abutments considered.

### 2.2. Clinical Assessment

The same professional, with extensive experience in implant dentistry, performed the clinical examination (M.A.B.). The considered parameters are described as follows. Prosthetic evaluations: (i) loosening of the fixation screw, (ii) fracture of the screw and (iii) the number of times the patient had some type of complication after the installation of the prostheses were evaluated; and biological evaluations (the band of keratinized mucosa was measured with a periodontal probe): (i) without keratinized mucosa (KM), (ii) 1 mm or less, (iii) between 1 and 2 mm and (iv) greater than 2 mm of KM width. The width of the KM was measured using the narrowest distance between the mucosal margin and the buccal mucogingival junction of each implant, using the visual and functional aspects as a parameter to identify differences in color, texture and mobility between keratinized and non-keratinized mucosa [18]. Finally, the presence (i) or absence (ii) of mucositis was evaluated [19].

### 2.3. Radiographic Evaluation

For the radiographic control and evaluation, marginal bone loss was evaluated using a digital periapical film RVG First intraoral system (Trophy, Toulouse, France) and a portable IriX-ray DX 3000 device (Dexcowin, Seoul, Republic of Korea) to capture the radiograph image of each implant. A radiographic positioner was used to maintain better parallelism and standardization of the images. To perform marginal bone level measurements on each implant, all images were exported and analyzed using ImageJ software version 1.44 (National Institute of Mental Health, Bethesda, ML, USA).

A medical computer display (Sony Inc., Tokyo, Japan) with magnification of 10× of each image was used for radiographic image analysis and measurements. Firstly, the software calibration was performed to make each measure using the implant dimensions (in diameter and length) described in the clinical history of each patient. For each implant, two positions were considered, mesial (mMBL) and distal (dMBL) marginal bone level, and each measurement was performed twice in the same position and repeated 14 days later by the same professional (S.A.G.) to calculate the degree of operator error.

**Figure 2:**
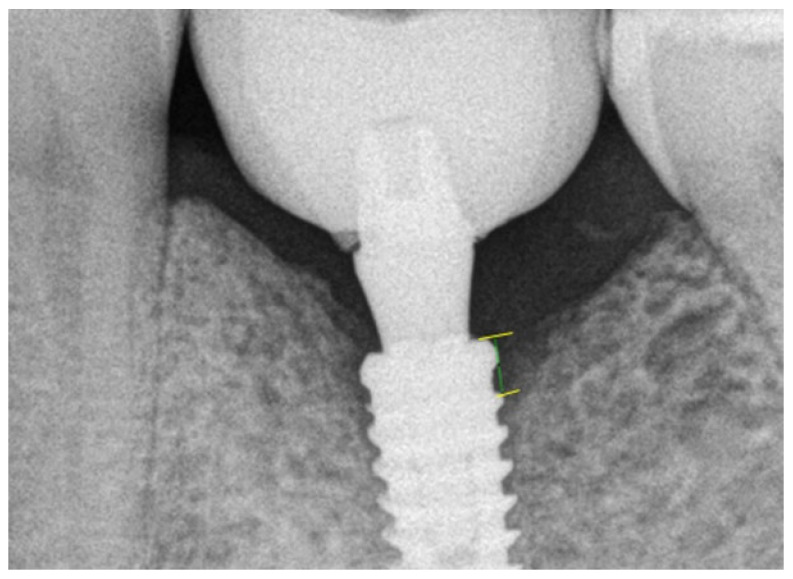
Representative image of the software showing the MBL measurement.

After having all data recorded and computed, the intra-examiner margin of error was calculated, which resulted in an average of 0.035 mm, indicating that the error was not statistically significant (*p* = 0.22, 95% CI). Figure 2 shows an image of the software calibration and the MBL measurement. An important observation is that all implants were installed at the crestal bone level.

### 2.4. Statistical Analysis and Sample Size Calculations

After analyzing the data using the Kolmogorov-Smirnov test, Levene’s homogeneity of variance test was verified for all data acquired. For bivariate analysis, Mann-Whitney U and Bonferroni multiple comparisons tests were applied. The correlation between implant diameter, implant length and time of the prosthesis in function versus MBL was tested using the Pearson test in both groups. All comparison analysis was performed using GraphPad Prism 8 software (GraphPad Software, San Diego, CA, USA). The level of significance was set at α = 0.05.

Due to the retrospective nature of this study, multiple linear regression analyses on overall bone loss in test and groups controls were performed separately. In the first situation, implant length, implant diameter and collar height were considered as independent variables, and the overall bone loss (mean of mesial and distal values) as the dependent variable at the 5% significance level.

## 3. Results

**Figure 3:**
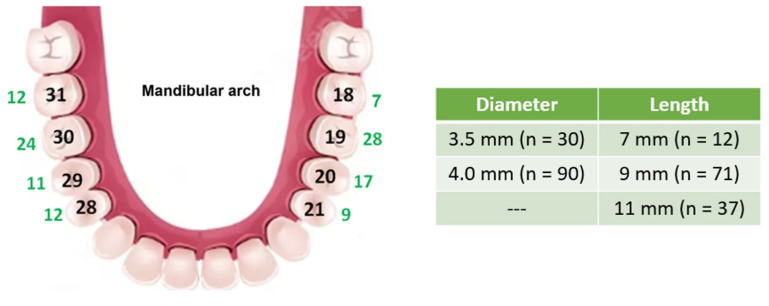
Details of the implant quantity distribution in the mandibular arch (green numbers) and table of implant dimensions (diameter and length) quantity.

79 patients, 31 men and 48 women aged between 35 and 82 (mean age of 58), were selected and invited for clinical evaluation based on their dental clinical history. Of these 79 patients, 28 were allocated to the control group and 51 to the test group. 120 MT implants were evaluated with a follow-up from 2 to 4 years. Figure 3 shows details of the implant distribution and dimensions.

**Figure 4:**
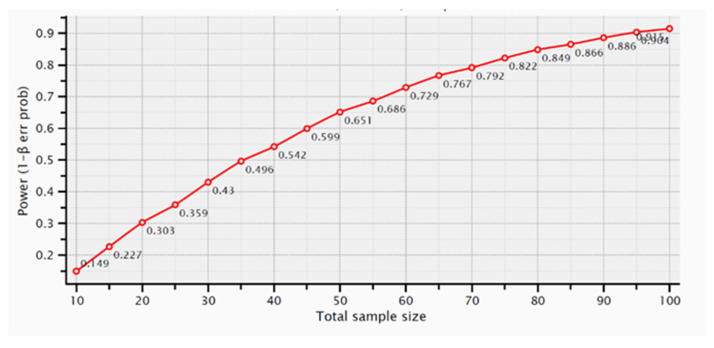
Samples number calculation for mesial MBL using *t*-tests—Means (Wilcoxon-Mann-Whitney test (two groups).

Regarding the number of analyzed samples, for mesial MBL, assuming mean and SD values of 1.2 ± 0.66 mm and 0.8 ± 0.4 mm, the calculated effect size would be 0.73 and the achieved power 0.95. The graph of Figure 4 shows the samples number calculation for mesial MBL.

**Figure 5:**
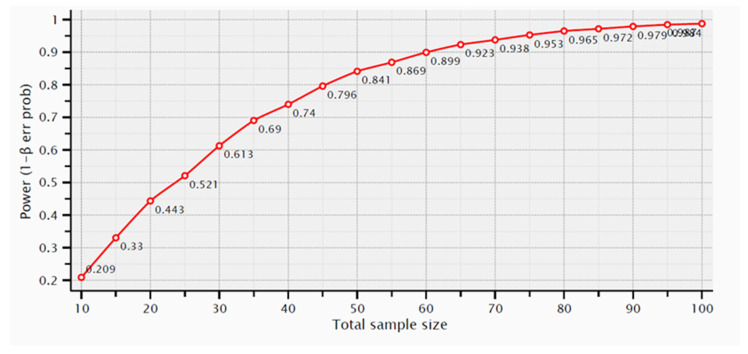
Samples number calculation for distal MBL using *t*-tests—Means (Wilcoxon-Mann-Whitney test (two groups).

For distal bone loss, assuming mean and SD values of 1.2 ± 0.64 mm and 0.7 ± 0.42 mm, with correspondent sample sizes of 80 and 40 dental implants, respectively, the calculated effect size would be 0.92 and the achieved power 0.99. The graph of Figure 5 shows the samples number calculation for distal MBL.

**Figure 6:**
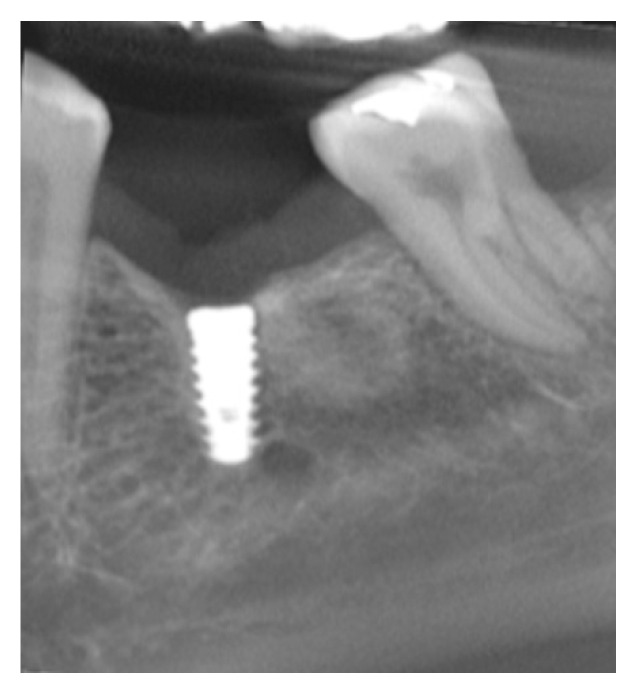
Radiographic image of a fractured abutment.

Eighty rehabilitations had castable UCLA abutments (test group), while another 40 had machined solid abutments (control group). All implants that were evaluated survived after the installation of the prosthesis. Regarding the mechanical problems related to the clinical histories, the control group showed two fractured abutments (5%) and no abutment loosening. Figure 6 shows a radiographic image of a case with a fractured abutment. In contrast, the test group showed no abutment fracture, but nine loosening screws (11.3%) were found.

After applying Normal and Equal Variance tests, a non-parametric test (General Linear Model) was chosen. For mesial MBL, the difference in the mean values among the different levels of abutment type was greater than expected by chance after allowing for effects of differences in transmucosal portion (TM) height (*p* = 0.001). Furthermore, the difference in the mean values among the different levels of TM height was greater than expected by chance after allowing for effects of differences in abutment type (*p* < 0.001). However, the effect of different levels of abutment type does not depend on what level of TM height was present. There was no statistically significant interaction between abutment type and TM height (*p* = 0.873) (Table 1).

For distal MBL, the difference in the mean values among the different levels of abutment type was greater than expected by chance after allowing for the effects of differences in TM height (*p* < 0.001). Additionally, the difference in the mean values among the different levels of TM height was greater than expected by chance after allowing for effects of differences in abutment type (*p* < 0.001). However, the effect of different levels of abutment type does not depend on what level of TM height was present. There was no statistically significant interaction between abutment type and TM height (*p* = 0.434). Then, all multiple pairwise comparisons on mesial and distal bone loss among groups were performed with the Bonferroni test, and can be seen in Table 2.

Then, all pairwise multiple comparisons on mesial and distal bone loss among groups were performed with the Bonferroni test, and can be seen on Table 3. In the test group (UCLA abutment), collar height was the only variable to account for the ability to predict MBL (*p* = 0.019). In the control group (solid abutment), collar height was the only variable to account for the ability to predict MBL (*p* = 0.005)

**Figure 7:**
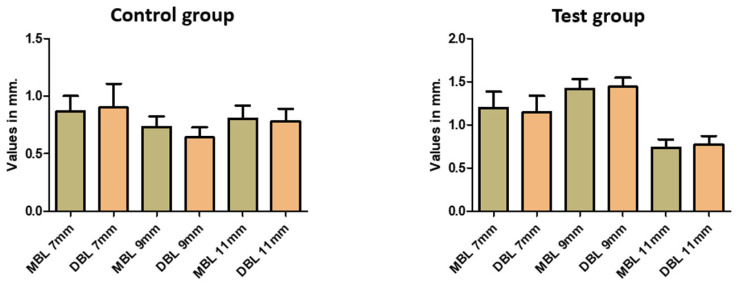
Marginal bone loss (mesial [MBL] and distal [DBL]) was compared among the different implants’ lengths.

Keratinized mucosa was considered thin or absent in 19 implants in the control group (47.5%) and 42 in the test group (52.5%). Mucositis was found in 11 implants in the control group (27.5%) and 27 in the test group (33.8%). A positive correlation was observed between the width of keratinized mucosa and mucositis (r = 0.521, *p* = 0.002). The overall mean marginal bone loss was 1.03 ± 0.62 mm, ranging from 0 to 3.2 mm. Figure 7 shows each implant length’s mean and standard deviation distribution of the marginal bone level (mMBL and dMBL). Table 4 shows the statistical comparison of the MBL values between the different implant lengths, and Table 5 shows the statistical comparison of the MBL intergroup at the same implant length.

**Figure 8:**
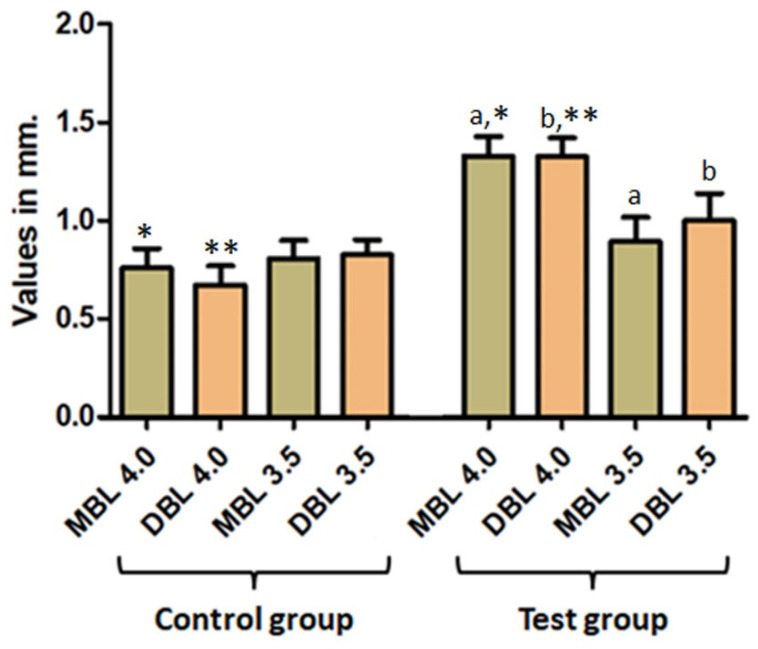
Relationship between implants’ diameter and marginal bone loss. a or b = intragroup statistically significant result (*p* < 0.05); * or ** (intergroup analysis) = *p* < 0.05.

Regarding the implant diameter comparison, it is important to highlight the statistical significance found for marginal bone loss, which was greater in the test group for standard implants (ø 4.0 mm) than the narrow platform (ø 3.5 mm), and the similar diameter in the control group. Figure 8 presents the data and statistical comparison between the values obtained in each group.

Considering marginal bone loss versus the three parameters (implant diameter, implant length and time of the prosthesis in function), no correlation was observed in either case.

## 4. Discussion

This present retrospective clinical study provides valuable insights into the clinical outcomes of different abutment types used for implant-supported restorations, comparing the performance of castable versus machined abutments. They were installed in 79 patients with 120 Morse taper implants involved and rehabilitated with unit crowns. This interface (Morse taper) presents better crestal bone maintenance and stability than hexagonal connections, with lower peri-implant bone loss [20,21,22]. It is essential to understand that the supracrestal soft tissue acts against bacteria that can be present in possible microleakage, and is closely related to bone remodeling [21,22]. Then, to reduce this variable, our study used only internal cone connection implants (Morse-taper), working on the platform switching concept. The literature has shown that using a smaller abutment’s diameter than the implant platform results in lower statistically significant values and more stability [23].

Our results permitted us to accept the null hypothesis proposed due to the general similarity of the data acquired. This fact is in line with those provided by Esposito et al.’s (2021) [24] results, where the authors suggested very similar short-term clinical outcomes, comparing both types of abutments and with other in-vitro studies [25,26,27,28,29]. It obtained a 100% implant survival rate, which agrees with the findings demonstrated by Borges et al. (2020) [30]. Nonetheless, there was no abutment fracture in the test group, but 9 cases presented loosening, while the control showed two fractured abutments and no abutment loosening. These findings suggest that castable UCLA abutments may be more fracture-resistant than machined solid abutments, but may be more prone to screw loosening. Other studies analyzed the clinical outcomes of different abutment types used for implant-supported restorations. In a retrospective cohort study of up to 18 years, Teigen and Jokstad (2012) found no significant difference in implant survival rates between castable and gold alloy abutments [31]. Otherwise, Pieri et al., (2013) [23] reported higher fracture rates for prefabricated abutments than castable abutments. The findings of the current study are consistent with the latter study.

The positive correlation between the width of keratinized mucosa and mucositis highlights the importance of maintaining adequate soft tissue support around implants to prevent peri-implant inflammation. The mean marginal bone level was 1.03 mm, ranging from 0 to 3.2 mm. They were within an acceptable range. However, there was no observed correlation between marginal bone loss and years of the prosthesis in function. This could be due to factors such as sample size, patient compliance or other variables not accounted for in the study. Furthermore, it is essential to highlight the statistical significance found for marginal bone loss, which was greater in the test group for standard implants (ø 4.0 mm) compared to the narrow platform (ø 3.5 mm) and the similar diameter in the control group. Additionally, the longer implant (11 mm) had a more significative marginal bone loss (mesial and distal) in the test group (castable UCLA) than 7 mm- and 9 mm-height implants, a fact proven by Fernandes et al. (2022) [32].

It is also concerning that KM was thin or absent in 19 implants in the control group (47.5%) and 42 in the test group (52.5%), respectively 27.5% and 33.8% associated with mucositis. This highlights the importance of sufficient keratinized mucosa for implant success. This result shows that the KM width was positive and inversely correlated with peri-implant mucositis, suggesting a greater KM should be considered in sites that will receive and already have implants. This fact agrees with the results of Ravidà et al. (2022) [33], who showed that KMW < 2 mm might be a risk factor for developing peri-implant diseases, even though this fact is dependent on other site-specific characteristics, such as mucosa thickness, supracrestal tissue height, peri-implant bone thickness, pocket depth and superstructure crown design. The results of this study are consistent with previous research that has shown the importance of keratinized mucosa in maintaining implant stability and preventing mucositis [34,35]. The findings also suggest that castable UCLA abutments may be advantageous in reducing the risk of abutment fracture compared to machined solid abutments. However, this may still increase the risk of screw loosening.

Moreover, according to the material used in the rehabilitation, it is possible to highlight the occurrence of galvanic currents. This is due to the titanium implant material and cobalt-chromium used in the abutment. Through galvanic analysis, the literature shows that cp Ti (without surface treatment) behaved as an anode; after acid treatment, it has a cathodic behavior concerning the CrCoMo alloy. This alloy (CoCrMo) has a lower corrosion resistance than titanium alloys. Moreover, the same study reported the highest value of galvanic current found in the cpTiG4 acid etched in contact with CoCrMo, and the lowest current in the nanostructured cpTi in contact with the same alloy [36].

### Study Limitations

It is important to note that the study had limitations: (i) the short-medium follow-up period (between 2 and 4 years) and the small sample size (79 individuals, mean age of 58); (ii) it is a retrospective study; (iii) lack of differentiation of radiopacity between different abutments used, once the assessor was focused on the evaluation of bone levels; (iv) even that all implants were in the posterior area, different patterns of chewing can be observed; (v) different implants’ diameters and height, as well as crown design and size; and (vi) important factors that may affect implant success, such as occlusal forces and implant position, not being considered in this study. Otherwise, the results of this investigation may be transmitted with confidence to a broader population. However, future studies with extended follow-up periods of up to 10 years and larger sample sizes are needed to confirm these findings.

## 5. Conclusions

Within the limitations of this study, our results suggest that careful consideration should be given to the type of abutment used, and sufficient keratinized mucosa around the implant must be considered to ensure the best possible outcome. UCLA-type abutments are viable for rehabilitating implants with Morse taper connections, suggesting a lower fracture risk. Further research is necessary to confirm these findings and thoroughly evaluate the clinical performance and long-term outcomes.

## Figures and Tables

**Table 1:** General Linear Model for mesial bone loss.

Source of Variation	DF	SS	MS	*p*-Value
Abutment type	1	3.549	3.549	0.001
Collar height	1	3.999	3.999	<0.001
Interaction	1	0.00825	0.00825	0.873
Residual	116	37.541	0.324	
Total	119	45.776	0.385	

DF, degrees of freedom; SS, sum of squares; MS, mean square.

**Table 2:** General Linear Model for distal bone loss.

Source of Variation	DF	SS	MS	*p*-Value
Abutment type	1	6.474	6.474	<0.001
Collar height	1	3.816	3.816	<0.001
Interaction	1	0.178	0.178	0.434
Residual	116	33.647	0.290	
Total	119	45.219	0.380	

DF, degrees of freedom; SS, sum of squares; MS, mean square.

**Table 3:** Marginal bone loss (mean ± SD values).

	Abutment Type			
	Solid (*n* = 40)		UCLA (*n* = 80)	
Collar Height	Mesial	Distal	Mesial	Distal
1.5 (*n* = 49)	1.02 ± 0.46 ^a,c,e^	0.88 ± 0.47 ^g,i^	1.37 ± 0.51 ^a,b,e^	1.46 ± 0.48 ^f,i^
2.5 (*n* = 71)	0.61 ± 0.33 ^c,d^	0.57 ± 0.34 ^g,h^	1.00 ± 0.71 ^b,d^	0.99 ± 0.66 ^f,h^

The same small letters represent statistically significant differences. Two-way ANOVA and Bonferroni tests: ^a^, *p* = 0.001; ^b^, *p* = 0.004; ^c^, *p* = 0.027; ^d^, *p* = 0.007; ^e^, *p* = 0.047; ^f^, *p* < 0.001; ^g^, *p* = 0.085; ^h^, *p* = 0.002; and ^i^, *p* < 0.001. *p*-values < 0.05 are statistically significant.

**Table 4:** Marginal bone level (mesial and distal) compared among the different implants’ lengths. Intragroup analysis.

Comparison	Control Group (*p*)	Test Group (*p*)
m-MBL 7 mm × 9 mm	0.4546	0.6504
d-MBL 7 mm × 9 mm	0.2423	0.3020
m-MBL 7 mm × 11 mm	0.5805	0.0351 *
d-MBL 7 mm × 11 mm	0.5137	0.0115 *
m-MBL 9 mm × 11 mm	0.8422	0.0005 *
d-MBL 9 mm × 11 mm	0.2135	0.0005 *

m-MBL = mesial marginal bone level; d-MBL = distal marginal bone loss; *p* = *p*-value; * = statistically significant result.

**Table 5:** Marginal bone level (mesial and distal) compared between implants with the same lengths. Difference intergroup.

Comparison (Control × Test)	*p*-Value
m-MBL 7 mm vs. 7 mm	0.3484
d-MBL 7 mm vs. 7 mm	0.7773
m-MBL 9 mm vs. 9 mm	0.0003 * (lower in control)
d-MBL 9 mm vs. 9 mm	<0.0001 * (lower in control)
m-MBL 11 mm vs. 11 mm	0.8182
d-MBL 11 mm vs. 11 mm	0.9755

m-MBL = mesial marginal bone level; d-MBL = distal marginal bone loss; * = statistically significant result.

## Data Availability

All data generated or analyzed during this study are included in this published article.
